# An investment case analysis for the prevention and treatment of adolescent mental disorders and suicide in England

**DOI:** 10.1093/eurpub/ckad193

**Published:** 2023-11-24

**Authors:** Angela Jackson-Morris, Christina L Meyer, Antony Morgan, Rachel Stelmach, Leah Jamison, Candace Currie

**Affiliations:** Center for Global Noncommunicable Diseases, RTI International, Durham, NC, USA; Center for Global Noncommunicable Diseases, RTI International, Durham, NC, USA; Glasgow Caledonian University, London, UK; International Development Group, RTI International, Washington, DC, USA; Center for Global Noncommunicable Diseases, RTI International, Durham, NC, USA; Glasgow Caledonian University, London, UK

## Abstract

**Background:**

Adolescent mental health (AMH) needs in England have increased dramatically and needs exceed treatment availability. This study undertook a comparative assessment of the health and economic return on investment (ROI) of interventions to prevent and treat mental disorders among adolescents (10–19 years) and examined intervention affordability and readiness.

**Methods:**

Interventions were identified following a review of published and grey literature. A Markov model followed a simulated adolescent cohort to estimate implementation costs and health, education, and economic benefits. Intervention affordability was assessed, comparing annual cost per adolescent with NHS England per capita spending, and an expert panel assessed intervention readiness using a validated framework.

**Results:**

Over 10- and 80-year horizons, interventions to treat mild anxiety and mild depression were most cost-effective, with the highest individual lifetime ROI (GBP 5822 GBP 1 and GBP 257: GBP 1). Preventing anxiety and depression was most affordable and ‘implementation ready’ and offered the highest health and economic benefits. A priority package (anxiety and depression prevention; mild anxiety and mild depression treatment) would avert 5 million disability-adjusted life-years (DALYS) and achieve an ROI of GBP 15: GBP 1 over 10 years or 11.5 million DALYs (ROI of GBP 55: GBP 1) over 80 years.

**Conclusion:**

The economic benefits from preventing and treating common adolescent mental disorders equivalent to 25% of NHS England’s annual spending in 2021 over 10 years and 91% over 80 years. Preventing and early treatment for anxiety and depression had the highest ROIs and strong implementation readiness.

## Introduction

Adolescent mental health (AMH) is critically important to individuals, families and policymakers grappling with decisions related to health, economic and social outcomes. Mental disorders are clinically significant disturbances in an individual’s cognition, emotional regulation or behaviour, usually associated with distress or impairment in important functional areas.^1^ An estimated 50% of lifetime mental disorders emerge before age 14, 75% by age 24^2^; early onset is associated with worse lifetime clinical outcomes.^3^ Global projections indicate that associated costs will double between 2011 and 2030, with two-thirds of this burden attributable to indirect costs such as productivity loss, mortality and disability.^4^ Combined, these impacts make a compelling case for greater investment in preventing and providing treatment for adolescents who experience mental disorders.

In 2017, one in eight young people aged 5–19 in England experienced a mental disorder.^5^ UK hospital admissions for self-harm for ages 9–17 increased 22% between 2020 and 2022.^6^ Reports indicate extensive waiting times for diagnosis and starting treatment, insufficient capacity to assess all adolescents experiencing mental disorders, and an urgent need for greater prevention.^7^^,^^8^ Government funding commitments and plans included a 1.4 billion commitment to transform child and AMH services in 2015,^9^ a 2017 green paper, and the 2019 NHS Long-Term Plan set out an intention to develop school and college support services. However, the COVID-19 pandemic and lockdowns, school and youth facility closures, and reduced mental health service capacity have squeezed resources.^10^^,^^11^

The National Institute of Health and Care Excellence produces evidence-based guidelines, including consideration of costs and benefits; however, guidelines focus on selected, specific mental disorders^12^ and use existing economic evidence rather than *de novo* analyses.^13^ To date no comparative assessment of cost-effectiveness across interventions to prevent and treat a range of high-impact adolescent mental disorders has been conducted. In a high-inflation fiscal context where government and civil society expenditure is constrained, this study quantified the costs and benefits of interventions to prevent or treat anxiety, depression, bipolar disorder and suicidal behaviour among adolescents in England and to identify the interventions that will produce the strongest return on investment (ROI).

The study also assessed key aspects of intervention ‘implementability’ to inform planning and capacity development. This included calculating intervention relative affordability and insight into system and sector implementation readiness.

## Methods

### Model overview

The study adapted a model developed for a global AMH investment case.^14^ The Markov model, programmed in R (version 4.2.1), quantified the costs and expected benefits of interventions to prevent and/or treat anxiety, bipolar disorder, depression and suicide among adolescents (ages 10–19 years).^14^ It estimated the intervention implementation costs and their associated health, education and economic benefits. Costs and benefits were discounted at an annual rate of 3% (varied between 0% and 5% in the sensitivity analysis) and expressed in 2021 GBP.^15^

### Model population and inputs

The model followed a simulated cohort of all adolescents in England (2020 population). The cohort was divided into groups reflecting the prevalence of acute anxiety disorders, major depressive disorder and bipolar disorder by age and sex, according to the 2019 Global Burden of Disease (GBD) Study.^16^ These disorders represent over 60% of the disease burden attributable to mental disorders amongst adolescents in England.^16^

The model applied the probability of the cohort transitioning between different mental health states (e.g. full health, acute episode, asymptomatic) over an 80-year period to estimate health impact over time. The initial health state was based on disorder prevalence.^16^ The probabilities of moving between states were drawn from peer-reviewed literature and global databases.^14^^,^^16^ The interventions then influenced an individual’s probability of moving between these states, reducing the risk of developing disorders or improving the likelihood of recovery.

The model captured the lifetime effects of interventions implemented during adolescence defined by the World Health Organization as those between 10 and 19 years of age.^17^ A 10-year time horizon also examined the short-term effects after the entire model cohort aged out of adolescence. Once an adolescent became an adult in the model, no additional interventions were applied. However, any short-term effects of interventions implemented during adolescence remained in effect based on the timeframe indicated by the evidence.

To estimate the overall economic impact of mental disorders, the model evaluated the impact on health, education and employment outcomes. To quantify health impacts, it calculated the impact on morbidity and mortality using disability weights established in GBD 2019.^12^ The model accounted for background morbidity from conditions other than the mental disorders of interest and did not include asymptomatic cases.

The impact of mental disorders on educational attainment in adolescence was assessed using the probability of completing educational milestones. The probabilities of completing certain levels of education by sex and age were drawn from ILOSTAT and applied an effect size for the estimated effect of each disorder on completing the given level.^18^

Impacts of educational attainment and health upon long-term employment and earnings were assessed using a human capital approach. The analysis valued the impact of additional years of education on the probability of employment and on expected wages. It assumed no one under the age of 15 was employed and accounted for the short-term effects of an acute mental disorder episode on expected workforce productivity in terms of absenteeism and presenteeism.

### Identifying interventions

The study included preventative and treatment interventions to address anxiety, depression, bipolar disorder, and suicide among adolescents in various settings, including schools, health facilities, and digitally. Interventions were identified from peer-reviewed literature, grey literature, and interviews with national experts (Supplementary table S1).

Additionally, semi-structured qualitative interviews were conducted with six researchers and practitioners from academia, non-governmental organizations and government agencies with background in AMH psychology, psychiatry and public health in England. Experts were selected based on their knowledge of the evidence on AMH interventions and their understanding of the current interventions in place and systemic gaps. Interview questions related to the burden of mental disorders, national priorities, service availability and promising (evaluated) interventions. The protocol was reviewed by the RTI International Institutional Review Board and determined to be Not Human Subjects Research.

### Extracting data from selected interventions

Information on intervention design, target population, evaluation methodology, outcome measures, effect size and costs or cost-effectiveness was extracted. Within each disease area and intervention category, interventions were prioritized by effectiveness. Where interventions for a given area were unavailable from the evidence base for England, effective interventions from the global literature were included. Interventions that did not assess the impact on the incidence of developing or recovering from a disorder were mapped to global meta-analyses for similar interventions from which replacement effect sizes were drawn.

### Selecting interventions for the model

A group of national experts provided feedback on the short-listed interventions regarding intervention appropriateness and feasibility in the national context and assumptions related to estimated coverage, delivery mode and setting. Prevention interventions included universal prevention of anxiety and depression; universal suicide prevention; and indicated prevention of suicide. Treatment interventions focused on mild anxiety; moderate and severe anxiety; mild depression; moderate and severe depression; and bipolar disorder. Baseline coverage levels were determined using estimates reported in grey literature^7^^,^^19^^,^^20^ and target coverage levels drawn from the national strategy (Supplementary table S2).^21^ The analysis focused on the impact of improving coverage of these illustrative interventions rather than the impact of the entire landscape of interventions currently implemented in England.

### Costing approach

Due to the limited availability of economic evaluations of AMH interventions in England and globally, intervention ingredients were extracted from the identified studies to build cost estimates for these interventions. This methodology was consistent with the approach of Stelmach et al.^14^ A standard list of resource needs and unit quantities was developed for each intervention based on intervention descriptions in their source documents (Supplementary tables S3 and S4).

### Outcome indicators

The analysis calculated the number of disability-adjusted life-years (DALYs) averted by each intervention to evaluate the health effects of implementing AMH interventions. DALYs represent the composite of premature mortality (years of life lost) and the prevalence and severity of ill health (years lived with disability) attributable to a disease or injury.^22^^,^^23^ Cost per DALY averted was used to compare intervention cost-effectiveness.^24^

The economic evaluation evaluated each intervention by its ROI, or net economic benefit minus cost, divided by cost. ROI represented the expected number of GBP returned to the economy for every GBP 1 invested. The analysis additionally examined a priority package of interventions with the highest lifetime ROIs.

A secondary outcome indicator was net benefits—the difference in the change in the economic benefits and the change in economic costs incurred by strengthening intervention coverage. Whereas ROI controlled for different levels of need and coverage of the interventions by dividing by costs, the net benefit was a gross sum reflecting intervention scale and coverage.

### Sensitivity analysis

A probabilistic sensitivity analysis (PSA) was conducted for each intervention or set of interventions to generate a 90% uncertainty interval (90% UI) for the outcomes of interest using a Latin hypercube sampling approach.^14^

To compare the difference in outcomes by sex, the study conducted a linear mixed-effects analysis of the effect of sex on the outcome of interest with sex as a fixed effect plus a random effect for hypercube sample row to control for repeated measures of the same PSA sample. A likelihood ratio test comparing this model with a model of only the random effects by hypercube sample row was then conducted; results were considered significantly different by sex at a level α = 0.05.

### Implementability assessment

A second-stage analysis examined the implementability of the selected interventions, specifically their affordability and the implementation readiness of sectors and systems. Relative affordability was calculated by comparing each intervention’s annual cost per adolescent to NHS England’s annual expenditure per capita in 2021–22 of GBP 2409, reported in 2021 GBP.^25^

Readiness to implement interventions was assessed using an online scoring exercise completed by ten purposively identified individuals with experience planning, assessing, or implementing AMH interventions. Questions were selected from implementation-related factors identified in the ‘Consolidated Framework on Implementation Research’ (Supplementary table S5).^26^ Respondents rated each intervention for all implementation factors using a Likert scale from 1 (lowest) to 5 (highest). The sum of all respondents’ scores for each intervention was used to rank interventions from highest (most ready) to lowest (least ready).

## Results

### Health impacts

The adolescent anxiety and depression prevention intervention averted 7.3 million DALYs among England’s adolescents over a lifetime, and the mild anxiety treatment and mild depression treatment averted over 4.2 million DALYs and over 1.3 million DALYs, respectively (table 1). Although the suicide prevention interventions averted fewer DALYs compared to the other interventions, school-based suicide prevention can avert 65 000 DALYs over the cohort’s lifetime. Whereas females were expected to experience nearly 60% of the averted disease burden by implementing anxiety, depression and bipolar disorder interventions, males were expected to experience 70% of the averted lifetime disease burden by suicide prevention interventions.

**Table 1:** DALYs averted by intervention over 10- and 80-year time horizons (in hundred thousands), by sex

Intervention	10 years			80 years		
	Total	Female	Male	Total	Female	Male
	(90% UI)	(90% UI)	(90% UI)	(90% UI)	(90% UI)	(90% UI)
Mild anxiety treatment	30.3	17.9	12.4	42.4	24.7	17.7
	(22.5–38.7)	(13.3–22.9)	(9.2–15.9)	(27.7–60.9)	(16.2–35.4)	(11.5–25.5)
School-based anxiety and depression prevention	18.8	10.9	8.0	73.1	41.7	31.4
	(14.6–24.1)	(8.4–13.9)	(6.2–10.1)	(44.8–117.0)	(25.5–67.1)	(19.4–50.2)
Mild depression treatment	10.5	6.2	4.3	13.2	7.7	5.6
	(4.3–16.4)	(2.6–9.7)	(1.8–6.8)	(5.6–21.2)	(3.2–12.2)	(2.3–9.0)
Moderate-severe depression treatment	1.0	0.6	0.4	1.2	0.7	0.5
	(0.2–1.7)	(0.1–1.0)	(0.1–0.7)	(0.3–2.1)	(0.2–1.2)	(0.1–0.9)
Bipolar disorder treatment	0.7	0.4	0.3	0.7	0.4	0.3
	(0.2–0.9)	(0.1–0.5)	(0.1–0.4)	(0.2–0.9)	(0.1–0.5)	(0.1–0.4)
Moderate-severe anxiety treatment	0.5	0.3	0.2	0.7	0.4	0.3
	(0.1–1.5)	(0.0–0.9)	(0.0–0.6)	(0.1–2.1)	(0.1–1.2)	(0.0–0.9)
School-based suicide prevention	0.1	0.0	0.1	0.6	0.2	0.5
	(0.1–0.2)	(0.0–0.1)	(0.0–0.1)	(0.3–1.1)	(0.1–0.3)	(0.2–0.8)
Hospital-based suicide prevention	0.0	0.0	0.0	0.0	0.0	0.0
	(0.0–0.0)	(0.0–0.0)	(0.0–0.0)	(0.0–0.0)	(0.0–0.0)	(0.0–0.0)
Priority package^a^	49.6	29.2	20.4	114.8	66.2	48.7
	(39.3–59.6)	(23.1–35.0)	(16.2–24.6)	(76.1–164.2)	(43.9–94.6)	(32.4–69.6)

a The priority package included the (i) school-based anxiety and depression intervention; (ii) mild anxiety treatment intervention; and (iii) mild depression treatment intervention.

A priority package of AMH interventions (anxiety and depression prevention; mild anxiety and mild depression treatment) could reduce DALYs by 5 million in the 10-year post-implementation period and 11.5 million DALYs over the model cohort’s lifetime.

### Intervention cost-effectiveness

**Figure 1:**
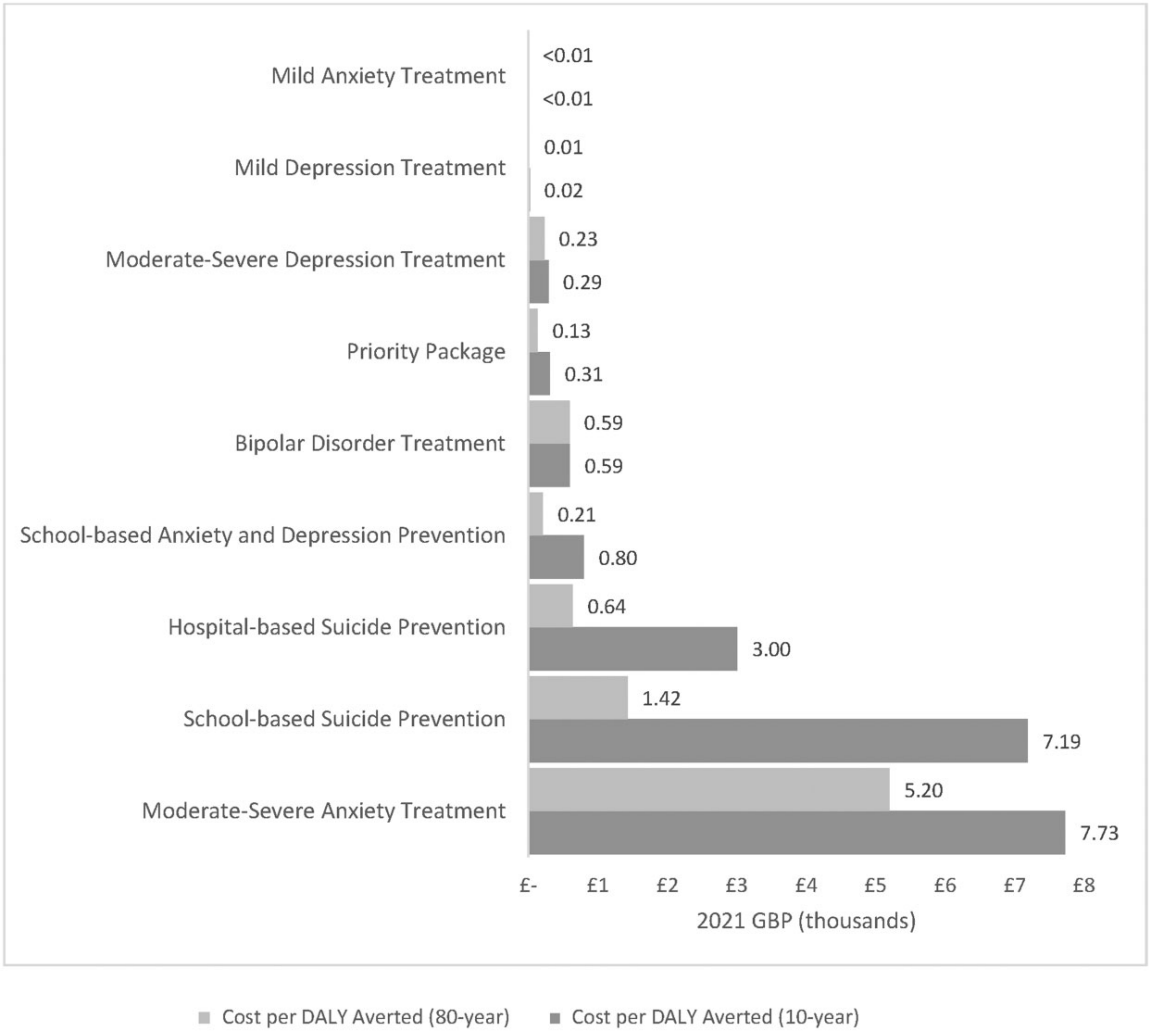
The cost per DALY averted in 2021 GBP at 10- and 80-year time horizons for individual adolescent mental health interventions and the priority intervention package. The mild anxiety treatment intervention has the lowest cost per DALY averted over both time horizons while the intervention to treat moderate-severe anxiety has the highest cost per DALY averted over the two time horizons

Figure 1 illustrates the cost per DALY averted at 10- and 80-year time horizons for individual interventions and the priority intervention package. Interventions for treating mild anxiety (GBP 1.3 per DALY at 10 years and GBP 0.93 at 80 years) and all levels of depression had the lowest individual cost per DALYs averted in contrast to the more costly suicide prevention and moderate-severe anxiety treatment interventions. The cost per DALY averted decreased over time as more DALYs were averted and benefits accrued, particularly for the prevention interventions. The priority package costs GBP 305 to avert each DALY over a 10-year time horizon and GBP 132 over an 80-year time horizon.

### Economic benefits

The treatment interventions for mild anxiety (5,921:1) and mild depression (257:1) had the strongest lifetime ROIs, followed by the prevention intervention for anxiety and depression (41:1) (table 2). The priority package comprising these interventions returned GBP 15 per GBP invested over a 10-year time horizon (table 2) with a total of GBP 34 billion in net benefits (table 3). Over an 80-year time horizon, the priority package returned GBP 55 per GBP invested with a total of GBP 122 billion in net benefits.

**Table 2:** ROI by intervention and sex over 10- and 80-year time horizons (2021 GBP)

Intervention	10 years			80 years		
	Total	Female	Male	Total	Female	Male
	(90% UI)	(90% UI)	(90% UI)	(90% UI)	(90% UI)	(90% UI)
Mild anxiety treatment	3536.4	3561.4	3498.2	5920.5	5822.2	6071.3
	(2283.6 to 4018.3)	(2302.9 to 4039.5)	(2261.0 to 3985.0)	(3207.9 to 7915.5)	(3157.5 to 7728.6)	(3289.9 to 8132.0)
School-based anxiety and depression prevention^a^	6.2	7.5	5.0	40.5	46.8	34.5
	(4.6 to 7.7)	(5.5 to 9.3)	(3.6 to 6.2)	(25.5 to 59.8)	(29.4 to 68.7)	(21.8 to 51.4)
Mild depression treatment	167.5	172.7	160.1	257.2	257.7	256.3
	(36.0 to 391.2)	(37.1 to 402.9)	(34.4 to 379.1)	(51.7 to 594.9)	(51.8 to 593.0)	(51.6 to 600.6)
Moderate-severe depression treatment	9.9	10.3	9.5	15.4	15.5	15.4
	(3.7 to 15.2)	(3.8 to 15.7)	(3.5 to 14.6)	(5.2 to 21.9)	(5.2 to 22.0)	(5.1 to 22.2)
Bipolar disorder treatment	4.1	4.2	4.1	4.2	4.3	4.1
	(0.9 to 5.3)	(0.9 to 5.3)	(0.9 to 5.2)	(0.9 to 5.4)	(0.9 to 5.4)	(0.9 to 5.3)
Moderate-severe anxiety treatment	0.0	0.0	0.0	0.5	0.5	0.5
	(−0.6 to 1.8)	(−0.6 to 1.9)	(−0.6 to 1.7)	(−0.5 to 3.1)	(−0.5 to 3.1)	(−0.5 to 3.0)
School-based suicide prevention	−0.4	−0.5	−0.3	0.9	0.2	1.6
	(−0.5 to −0.3)	(−0.6 to −0.4)	(−0.5 to −0.1)	(0.0 to 2.0)	(−0.3 to 0.8)	(0.3 to 3.0)
Hospital-based suicide prevention	0.0	−0.2	0.4	2.9	1.5	4.9
	(−0.2 to 0.3)	(−0.4 to −0.1)	(0.0 to 0.8)	(1.4 to 5.1)	(0.6 to 2.9)	(2.6 to 8.1)
Priority package	15.0	18.5	11.7	54.7	64.1	45.7
	(11.5 to 16.7)	(14.1 to 20.7)	(8.8 to 13.0)	(34.4 to 73.3)	(40.3 to 86.2)	(28.7 to 61.3)

a Significantly different ROIs based on sex (*P* < 0.05).

**Table 3:** Net benefits by intervention and sex over 10- and 80-year time horizons (2021 GBP in billions)

Intervention	10 years			80 years		
	Total	Female	Male	Total	Female	Male
	(90% UI)	(90% UI)	(90% UI)	(90% UI)	(90% UI)	(90% UI)
Mild anxiety treatment^a^	20.5	12.5	8.0	34.4	20.5	13.9
	(13.9 to 24.1)	(8.5 to 14.7)	(5.4 to 9.4)	(19.3 to 49.5)	(11.5 to 29.3)	(7.7 to 20.2)
School-based anxiety and depression prevention^b^	13.7	8.1	5.6	89.4	50.4	39.0
	(9.9 to 17.6)	(5.8 to 10.3)	(4.0 to 7.2)	(53.3 to 135.2)	(30.1 to 76.3)	(23.2 to 58.9)
Mild depression treatment	4.7	2.8	1.8	7.2	4.2	2.9
	(1.5 to 7.1)	(0.9 to 4.3)	(0.6 to 2.8)	(2.2 to 11.4)	(1.3 to 6.6)	(0.9 to 4.7)
Moderate-severe depression treatment	0.4	0.2	0.2	0.6	0.4	0.3
	(0.1 to 0.7)	(0.0 to 0.4)	(0.0 to 0.3)	(0.1 to 1.0)	(0.1 to 0.6)	(0.0 to 0.4)
Bipolar disorder treatment	0.3	0.1	0.1	0.3	0.1	0.1
	(0.1 to 0.3)	(0.0 to 0.2)	(0.0 to 0.2)	(0.1 to 0.3)	(0.0 to 0.2)	(0.0 to 0.2)
Moderate-severe anxiety treatment	0.0	0.0	0.0	0.3	0.2	0.1
	(−0.4 to 0.8)	(−0.2 to 0.5)	(−0.2 to 0.3)	(−0.3 to 1.3)	(−0.2 to 0.8)	(−0.1 to 0.5)
School-based suicide prevention	−0.1	0.0	0.0	0.1	0.0	0.1
	(−0.1 to 0.0)	(0.0 to 0.0)	(0.0 to 0.0)	(0.0 to 0.3)	(0.0 to 0.1)	(0.0 to 0.2)
Hospital-based suicide prevention	0.0	0.0	0.0	0.0	0.0	0.0
	(0.0 to 0.0)	(0.0 to 0.0)	(0.0 to 0.0)	(0.0 to 0.0)	(0.0 to 0.0)	(0.0 to 0.0)
Priority package^b^	33.5	20.2	13.3	121.7	69.8	51.9
	(25.1 to 37.9)	(15.2 to 22.9)	(10.0 to 15.0)	(74.9 to 169.0)	(42.9 to 96.4)	(32.1 to 72.5)

a Significantly different net benefits based on sex at the 80-year time horizon (*P* < 0.05).

b Significantly different net benefits based on sex at both 10- and 80-year time horizons (*P* < 0.05).

Moderate and severe depression treatment and bipolar disorder treatment had the next strongest ROIs over a lifetime. Compared with other interventions, the adolescent suicide prevention interventions modelled offered a smaller lifetime ROI, yet the school-based suicide prevention intervention still created a total net benefit equivalent to GBP 123 million. Treatment of moderate and severe anxiety was the only intervention where we could not say with 90% confidence that the benefits outweigh the costs as the lower bound of the ROI confidence interval is negative (90% UI: −0.5 to 3.1).

The ROI was significantly stronger for females who received the school-based anxiety and depression prevention intervention compared to males. Expanding AMH interventions in England would have a stronger impact on females. Both the priority intervention package and the anxiety and depression prevention intervention had significantly stronger ROIs among females compared to males (*P* < 0.05). Moreover, addressing the mental disorder burden among female adolescents would contribute to 57% of England’s total lifetime net benefits from implementing the priority package of AMH interventions (table 3).

### Affordability

The affordability analysis summarized the annual cost per adolescent who received each intervention in England by intervention, year of implementation, and share of NHS England and Improvement’s annual spending per capita in 2021–22 (Supplementary table S7).^27^ Intervention implementation costs were typically highest in the first year. Universal school-based prevention interventions were among the most affordable interventions, particularly the suicide prevention intervention, which had the lowest annual cost per adolescent recipient at <1% of the annual NHS spending per capita in the UK.^28^ The annual cost of universal prevention of anxiety and depression per adolescent recipient was equivalent to 3% of annual NHS spending per capita. Treating moderate and severe anxiety incurred an annual cost that exceeded annual per capita NHS England spending by more than 27% (GBP 3223 for the first year and GBP 3057 for subsequent years).

### Readiness

Based on the ranked total readiness scores, the AMH interventions rated as most ‘implementation ready’ were as follows: (i) indicated prevention of suicide, (ii) universal school-based prevention of suicide and (iii) universal school-based prevention of anxiety and depression (Supplementary table S8). Preventative interventions generally received the highest overall scores, with school-based suicide prevention and mild anxiety and depression prevention interventions highly ranked.

## Discussion

This study found that addressing the high and growing level of unmet needs among England’s adolescents offers lifelong health and economic benefits. It quantified the expected costs and benefits of a range of evidence-based interventions to prevent or treat anxiety, depression, bipolar disorder and suicide. Each assessed intervention was found to reduce the national disease burden and benefit adolescent mental wellbeing.

Existing evidence indicated that access and availability of support to protect young people’s mental health is a significant issue,^7^ while data also suggested that need may have increased during the COVID-19 pandemic,^11^ and possibly since then due to impacts of the ‘cost of living crisis’.^29^ Although prior cost-of-illness studies have explored the economic burden of AMH in England,^30^ this analysis is the first England-specific AMH investment case and offers a unique perspective on the health and economic benefits that can be gained by expanding access to prevention and treatment. Moreover, in a constrained fiscal context,^30^ this analysis’ identification of interventions with the highest health and economic returns meets the need for government and third-sector organizations to maximize the benefits that programmes and services can generate for adolescents and society.^31^

Interventions to treat mild cases of anxiety and, to a lesser extent, depression offered the highest ROI with minimal per-person implementation costs. This aligns with the *Disease Control Priorities, 3rd edition’s* recommendations.^31^ However, both interventions received lower implementation readiness scores compared to other interventions. This may be because the consulted experts ranked the need for treatment as relatively less acute than the unmet need for prevention—an emphasis seen in the wider literature.^32^^,^^33^ Preventing anxiety and depression in schools had a strong ROI and was one of the most affordable and implementation-ready interventions. The results indicate early intervention to address anxiety and depression among England’s adolescents (treating mild symptoms and delivering prevention programmes to whole age cohorts) as a critical area for investment.

The net economic benefits associated with implementing a priority package of interventions to prevent and treat mild cases of anxiety and depression over a 10-year horizon was equivalent to 25% of NHS England’s 2021–22 spending or over 91% of this annual spending over a ‘lifetime’ horizon of 80 years.^28^ The priority packages would avert 5.0 million DALYS and achieve an ROI of 15:1 over a 10-year period or avert 11.5 million DALYs with an ROI of 55:1 over a lifetime horizon. As females made up three out of every five cases of adolescent major depressive disorder and anxiety disorder in England,^16^ implementing this intervention package among girls offered a statistically greater number of net economic benefits. Nonetheless, positive ROIs for the priority intervention package were found for both sexes within a 10-year horizon, indicating that interventions should be inclusive.

Both suicide prevention interventions had the highest readiness scores of the assessed interventions—indicating both need and implementability. However, their implementation costs per DALY averted were higher and generated relatively fewer net economic benefits over the long term. The high readiness scores may have reflected the wider national focus on adolescent suicide following high-profile cases and campaigns by bereaved parents.^34^^,^^35^ Given the high demand and system readiness, it would be valuable to identify and evaluate more cost-effective interventions for adolescent suicide prevention than were available for this analysis.

Similarly, treating moderate and severe anxiety ranked highly on readiness yet had the highest implementation costs of all interventions, outweighing long-term benefits if implemented individually. As this intervention required psychologists or psychiatrists to directly administer treatment, strong investment in the more ‘upstream’ anxiety prevention and mild treatment interventions would be recommended to reduce the incidence of moderate and severe anxiety and demand for this more costly intervention.

Lastly, although school-based interventions were relatively affordable and rated highly on readiness, especially regarding infrastructure, culture, leadership support and ‘fit’, the competing priorities of other school initiatives and delivery pressures were identified as important to address. Prior research highlighted the need to increase school intervention delivery capacity, particularly with staff training and ringfenced time to manage students’ increased mental health needs.^36^

We believe this was the first comparative assessment of the cost-effectiveness of a range of interventions to prevent and treat various high-impact AMH issues in England. A major strength was that assessed interventions were based on examples already implemented or piloted in England. The implementability assessment added insight into each intervention’s relative affordability and readiness and identified areas where pre-implementation capacity development would be beneficial.

Several limitations should be noted. The authors recognized the importance of initiatives to promote positive mental health and hoped to reflect interventions across the continuum of care—including health promotion.^37^ However, limited robust evaluation and costing data from such initiatives impeded inclusion. Addressing this gap would enable future economic analysis across a broader intervention spectrum. The study assessed interventions for disorders that contribute a large proportion of the national disease burden, including bipolar disorder (high impact, albeit lower prevalence). Although other disorders such as conduct or eating disorders are of concern,^11^ these were not included to maintain comparability with the global model and parallel studies in other countries.

Intervention data from England were prioritized; however, when interventions for a disorder or intervention category were unavailable, the analysis included effective interventions from international studies, assuming that intervention effects are generalizable. Interventions identified as effective for preventing or improving symptoms but lacking evidence regarding the incidence of developing or recovering from a disorder were mapped to global meta-analyses for similar interventions, from which replacement effect sizes were drawn.^14^ Integrated service delivery or mental health awareness campaigns could improve the interventions’ ROI by improving efficiency (i.e. identifying additional adolescents who require the interventions) or reduce ROI by preventing disorders that the interventions would have otherwise addressed. However, without empirical evidence of this impact on the interventions, it cannot be captured in the model. Baseline coverage levels were determined using available estimates reported in grey literature. However, few peer-reviewed reports or studies detailed current coverage—an important area for further research to strengthen the national evidence base. Dissemination activity costs were not included as school-based or healthcare facility-based interventions are generally built into school/health facility processes. Study results are reported in GBP 2021, yet the UK’s inflation rate rose to an unprecedented 9.6% in 2022.^38^ Should inflation stabilize at this level, the results could be adjusted. As the model is based on a simulated cohort of all adolescents in England in 2020, there is likely a higher prevalence of mental health disorders as a result of the COVID-19 pandemic and wider mental health trends.^6^ The potential ROI and net benefits of the evaluated interventions, particularly preventative interventions, could be greater than the current estimates. If the disease burden was higher, the interventions’ cost per DALY averted would likely decrease based on economies of scale.

This analysis of the health and economic costs and benefits, affordability, and readiness of AMH interventions in England produced evidence that can inform the scale and type of investment needed to strengthen AMH provision. The majority of assessed interventions delivered significant health benefits to address unmet needs, although preventative and early treatment interventions offer the greatest health and economic ROI.

## Supplementary Material

ckad193_Supplementary_Data

## Data Availability

The data underlying this article will be shared on reasonable request to the corresponding author.
