# Supplementary material for: An investment case analysis for the prevention and treatment of adolescent mental disorders and suicide in England
Source: Eur J Public Health. 2023 Nov 24;34(1):107–13. doi: 10.1093/eurpub/ckad193 (PMC10843955; doi:10.1093/eurpub/ckad193)
Supplement: ckad193_Supplementary_Data [file ckad193_supplementary_data.zip › ckad193_Supplementary_Data/ejph-2023-05-om-0245-File003.docx]

**Supplementary Materials**

# **Peer-Reviewed Literature Search Protocol and Grey Literature Review**

The interventions included in the model were identified from peer-reviewed literature, unpublished grey literature documents, and interviews conducted with national experts. We first conducted a systematic literature search for studies of adolescent mental health interventions conducted in England. We searched PubMed, Embase, Web of Science, CAB Abstracts, Directory of Open Access Journals, Google Scholar, New York Academy of Medicine Grey Literature Database using the strategy presented in Table A1. All identified abstracts were screened for inclusion by two research team members (DH and EK). The included studies were then reviewed in full text form by two team members. All inclusion decisions were reached by consensus between these individuals.

**Table S1: Search Strategy**

| **Category** | **Terms** |
| --- | --- |
| Adolescents | adolescent* OR adolescence OR "young adult*" OR youth* OR teen* |
| Country | [Enter relevant country terms] |
| Interventions | intervention* OR program* OR policy OR policies OR outcome* OR impact* OR prevent* |
| Mental Health | "mental health" OR "mental disorder*" OR "mental illness*" OR "mentally ill" OR education OR employment OR anxiety OR suicide* OR suicidal OR depression OR bipolar |

We identified publications in the grey literature through systematic searches of the websites of government agencies (e.g., NHS England, National Institute of Health and Care Excellence, Office for Health Improvement and Disparities), non-governmental organizations (e.g., The King’s Fund, Centre for Mental Health, PLAN UK, Association for Young People’s Health), and multilateral agencies organizations, such as UNICEF. Grey literature was also identified from key informants’ recommendations.

**Description of Investment Case Interventions**

**Table S2** summarises the selected interventions and details the baseline program coverage and target population coverage goals for each intervention. Baseline coverage levels were determined using estimates reported in grey literature^1–3^ and target coverage levels drew from the NHS national strategy.^4^ The analysis focused on the impact of improving coverage of these illustrative interventions rather than the impact of the entire landscape of interventions currently implemented in England.

**Table S2. Description of Interventions and Baseline and Target Coverage Levels Among England’s Adolescent Population**

| **Intervention** | **Description** | **Baseline Coverage (Uncertainty Interval Range)** | **Target Coverage**  **(Uncertainty Interval Range)** |
| --- | --- | --- | --- |
| Prevention Interventions | | | |
| Universal prevention of anxiety and depression | The program was comprised of three components, including a universal school-based program for all students, a peer support component, and a mobile application. | 20%  (17% - 23%) | 85%  (72% - 98%) |
| Universal suicide prevention | All adolescents enrolled in a school would receive a school-based suicide prevention intervention. Students participated in in-person class sessions including lectures and interactive activities over a month. Sessions were led by a health worker, under a psychologist’s supervision. | 20%  (17% - 23%) | 85%  (72% - 98%) |
| Indicated prevention of suicide | Information sessions were offered to adolescents who attempted suicide and were treated for self-harm at a hospital. | 30%  (26% - 35%) | 70%  (60% - 81%) |
| Treatment Interventions | | | |
| Treatment of mild anxiety | Treatment of mild anxiety with an internet-based, guided self-help modules based on cognitive behavioural therapy (CBT) principles would be delivered using a specialized internet platform (computerized CBT). Adolescents had access to limited support from a mental health worker. | 10%  (9% - 12%) | 70%  (60% - 81%) |
| Treatment of moderate and severe anxiety | For adolescents with moderate and severe anxiety, or anxiety that was not sufficiently managed by computerized CBT, adolescents would receive individual CBT from a psychologist or psychiatrist, with the option of adding pharmacological treatment. | 50%  (43% - 58%) | 70%  (60% - 81%) |
| Treatment of mild depression | Treatment of mild depression with an internet-based, guided self-help modules based on cognitive behavioural therapy (CBT) principles was delivered using a specialized internet platform (computerized CBT). Adolescents have access to limited support from a mental health worker. | 10%  (9% - 12%) | 70%  (60% - 81%) |
| Treatment of moderate and severe depression | Adolescents with moderate and severe depression were provided with individual CBT. If patients did not respond to CBT alone, pharmacological care could be provided, with an increased dosage if the adolescent was still unresponsive. | 50%  (43% - 58%) | 60%  (51% - 69%) |
| Treatment of bipolar disorder | Family focused treatment for adolescents was a psychological intervention accompanied by pharmacotherapy. Sessions were administered by a psychiatrist and attended by adolescents and their family members. Adolescents in all stages of bipolar disorder received treatment with a mood stabilizer. In a depressive episode, adolescents received a mood stabilizer and anti-depressant medication. In a manic episode, adolescents received a mood stabilizer and an antipsychotic medication. | 30%  (26% - 35%) | 85%  (73% - 98%) |

The following provides a description of each of the investment case interventions, whether there was a pre-existing intervention evaluated in the UK or England, as well as the source the intervention’s effect size was extracted from.

## **Universal school-based prevention of anxiety and depression**

Description: The programme includes a universal (all pupils) school-based programme, peer support component, and a mobile application. The school-based programme is focused on building resilience and mental wellbeing, targeting both anxiety and depression symptoms. Lessons are delivered by school staff via 4 modules integrated into the Personal, Social and Health Education (PSHE) curriculum. Older pupils, provide advice and support to peers in drop-in sessions. The programme includes a mobile application (free download).

Reference(s): UK

- Mallika Punukollu, Caitlin Burns & Mafalda Marques (2020) Effectiveness of a pilot school-based intervention on improving Scottish students’ mental health: a mixed methods evaluation, International Journal of Adolescence and Youth, 25:1, 505-518, DOI: 10.1080/02673843.2019.1674167^5^
- Punukollu, M., Leighton, E.L., Brooks, A.F., Heron, S., Mitchell, F., Regener, P., Karagiorgou, O., Bell, C., Gilmour, M., Moya, N., Sharpe, H. and Minnis, H. (2020), SafeSpot: an innovative app and mental health support package for Scottish schools – a qualitative analysis as part of a mixed methods study. Child Adolesc Ment Health, 25: 110-116. https://doi.org/10.1111/camh.12375 ^6^

**Effect size: Stockings et al., 2016**^7^

| **Time point** | **Effect size (RR of developing disorder)** |
| --- | --- |
| Anxiety, post-intervention | RR 0.25 (0.10, 0.65) |
| Anxiety, 1-3 months | RR 0.20 (0.01, 4.21) |
| Anxiety, 6-9 months | RR 1.10 (0.45, 2.51) |
| Internalizing disorder, post- intervention | RR 0.39 (0.26, 0.59) |
| Internalizing disorder, 1-3 months | RR 0.33 (0.18, 0.61) |
| Internalizing disorder, 6-9 months | RR 0.47 (0.37, 0.6) |
| Depression, post-intervention | RR 0.41 (0.24, 0.69) |
| Depression, 1-3 months | RR 0.35 (0.24, 0.53) |
| Depression, 6-9 months | RR 0.47 (0.37, 0.60) |

## **Treatment of mild anxiety**

Description: Treatment of mild anxiety with an internet-based, guided self-help intervention can be delivered using a specialized internet platform. The intervention includes self-directed completion of modules based on cognitive behavioral therapy (CBT) principles (computerized CBT). Adolescents have access to limited support from a mental medical officer (3 calls of up to 5 minutes each). Modules should be completed over a minimum period of 6 weeks.

Reference(s): England

- [NICE Quality Standard](https://www.nice.org.uk/guidance/qs53), (Feb 2014)^8^

**Effect size: James et al, 2015.**^9^

| **Time point** | **Effect size (OR of disorder remission)** |
| --- | --- |
| Post intervention | OR 7.92, 95% CI 3.37 to 18.63 |
| 13.5 months mean follow up | OR 3.22, 95% CI 0.96 to 10.75, Z = 1.90, P = 0.06 |

## **Treatment of moderate to severe anxiety**

Description: For adolescents with moderate to severe anxiety or anxiety that is not sufficiently managed by computerized CBT, treatment is stepped up to a high intensity psychological intervention. This is one on one CBT, with the option of adding drug treatment (selective serotonin reuptake inhibitor – SSRI). Treatment is delivered by a psychologist or psychiatrist over 12 weekly 1-hour sessions.

Reference(s): England

- [NICE Quality Standard](https://www.nice.org.uk/guidance/qs53) - Last Updated Feb 2014^8^

**Effect size: James et al, 2015.**^9^

| Time point | Effect size **(OR of disorder remission)** |
| --- | --- |
| Post intervention | OR 7.92, 95% CI 3.37 to 18.63 |
| 13.5 months mean follow up | OR 3.22, 95% CI 0.96 to 10.75, Z = 1.90, P = 0.06 |

## **Treatment of mild depression**

Description: Treatment of mild depression with an internet-based, guided self-help intervention can be delivered using a specialized internet platform (computerized CBT). The intervention includes 8, 30–45-minute modules based on CBT principles that adolescents complete over 3 months. The 8 sessions are in a linear progression, with each session building on knowledge gained in previous sessions. Adolescents have access to limited support from a mental medical officer (3 calls of up to 5 minutes each).

Reference(s): England

- Abeles P, Verduyn C, Robinson A, Smith P, Yule W, Proudfoot J. Computerized CBT for adolescent depression ("Stressbusters") and its initial evaluation through an extended case series. *Behav Cogn Psychother*. 2009;37(2):151-165. doi:10.1017/S1352465808005067^10^

**Effect size: Oud et al., 2019 [CBT vs waitlist controls]**^11^

| Time point | Effect size |
| --- | --- |
| Post-treatment | RR remission 0.23 (0.13, 0.40) |
| Post-treatment | RR recovery 0.64 (0.42, 0.98) |
| Follow up (17–39 weeks) | RR depressed 0.37 (0.15, 0.92) |

## **Treatment of moderate to severe depression**

Description: For adolescents with moderate to severe depression or mild depression that is not sufficiently managed by computerized CBT, treatment is stepped up to a high intensity psychological intervention - 3 months of individual, one on one CBT . If improvement is not seen within 4-6 sessions anti-depressant medication can be prescribed. Treatment comprises 12 weekly 1-hour sessions. When a child or young person responds to treatment with fluoxetine, medication should be continued for at least 6 months after remission (defined as no symptoms and full functioning for at least 8 weeks); in other words, for 6 months after this 8-week period. Where antidepressant medication is to be discontinued, the drug should be phased out over a period of 6 to 12 weeks with the exact dose being titrated against the level of discontinuation/withdrawal symptoms.

Regional Reference(s): England

- [NICE Guideline](https://www.nice.org.uk/guidance/ng134) - Last updated June 2019^12^

**Effect size: Domino et al. 2008, Kennard et al. 2006, CBT + indicated medication use**^13,14^

| Time point | Effect size (OR of disorder remission) |
| --- | --- |
| Post intervention | OR 3.0, 95% CI 1.58 to 5.79 |

## **Treatment of bipolar disorder**

Description: Family focused treatment for adolescents (FFT-A) is a psychological intervention accompanied by pharmacotherapy. The intervention involves 16, 1-hour sessions delivered over 6 months (7 weekly psychosocial education sessions; 4 fortnightly communication enhancement sessions; 4 fortnightly problem-solving skills training sessions; 1 final session). Sessions are administered by a psychiatrist and attended by adolescents and family members. Adolescents in all stages of bipolar disorder receive treatment with a mood stabilizer. When in a depressive episode, adolescents receive a mood stabilizer and anti-depressant medication. When in a manic episode, adolescents receive a mood stabilizer and an antipsychotic medication. Mare accompanied by routine monitoring and appropriate laboratory tests.

Regional Reference(s): England

- Sharma, A., Glod, M., Forster, T., McGovern, R., McGurk, K., Barron Millar, E., ... & Le Couteur, A. (2020). FAB: First UK feasibility trial of a future randomised controlled trial of Family focused treatment for Adolescents with Bipolar disorder. *International Journal of Bipolar Disorders*, *8*(1), 1-14.^15^

**Effect size: Miklowitz et al., 2008**^16^

| **Time point** | **Effect size (HR for recovery)** |
| --- | --- |
| 2-year recovery from depressive symptoms | HR 1.85 (1.04, 3.29) |

## **Universal school-based prevention of suicide**

Description: All adolescents enrolled in a school will receive a school-based suicide prevention intervention. Students participate in 5-hours of in-person class sessions including lectures and interactive activities over 4 weeks. Sessions are led by a medical officer, under a psychologist’s supervision .

Regional Reference(s): N/A for England/UK

**Effect size: Wasserman et al., 2015**^17^

| **Time point** | **Effect size (OR for suicide attempt)** |
| --- | --- |
| 3 months, incident suicide attempt | OR 0.78 (0.42, 1.44) |
| 12 months, incident suicide attempt | OR 0.45 (0.24, 0.85) |

## **Indicated prevention of suicide**

Description: Intervention with individuals after a suicide attempt can effectively reduce the probability of future attempts. This intervention includes a 1-hour information session with adolescents treated for self-harm at a hospital. Adolescents then receive 9 brief follow-up contacts over 18 months. Information sessions and follow up contacts are conducted by mental health support workers under psychologist supervision.

Regional Reference(s): N/A for England/UK

**Effect size: Fleischman et al., 2008**^18^

| **Time point** | **Effect size (RR for probability of suicide)** |
| --- | --- |
| 18 months | RR 0.11 (0.02, 0.45) |

**Cost Components and Unit Costs**

Costs included medical officer and teachers’ salaries to deliver the interventions and complete associated training, medications, laboratory tests, information and communications technology equipment, online platform development and maintenance, and printed materials. Below is the list of unit costs reported in GBP 2021. The following also describes each intervention’s cost component unit count and description.

**Table S3. Cost descriptions and unit costs.**

| **Cost description** | **Traded/Non-traded** | **Unit Cost (GBP 2021)** | **Source** |
| --- | --- | --- | --- |
| Fluoxetine, per 20mg capsule | Traded | 0.06 | ^19^ |
| Sodium Valproate, per 500mg tablet | Traded | 0.18 | ^20^ |
| Olanzapine, per 10mg tablet | Traded | 0.28 | ^20^ |
| Therapist | Non-traded | 65.89 | ^21^ |
| Medical officer | Non-traded | 5.95 | ^21^ |
| Psychologist | Non-traded | 109.32 | ^21^ |
| Psychiatrist | Non-traded | 118.23 | ^21^ |
| Full blood count | Non-traded | 7.77 | ^22^ |
| Liver function test | Non-traded | 8.15 | ^22^ |
| Serum prolactin test | Non-traded | 60.57 | ^22^ |
| Teacher salary (starting) | Non-traded | 2.04 | ^23^ |
| Teacher salary (top of scale) | Non-traded | 2.70 | ^23^ |
| Materials printing (per page) | Non-traded | 0.08 | ^24^ |
| IT infrastructure | Non-traded | 40.50 | ^25^ |
| Transportation | Non-traded | 12.10 | ^26–31^ |
| Telephone | Traded | 30.66 | ^32^ |
| Phone call (per minute) | Traded | 0.07 | ^33^ |

## **Table S4. Cost components and units by adolescent mental health intervention**

| **Intervention** | **Ingredient** | **Units** | **Description** |
| --- | --- | --- | --- |
| Universal school-based prevention of anxiety and depression^5,6^ | Teacher salary | 8 hours | Teachers and peer mentors were trained through one day of face‐to‐face training and comprehensive information packs on the program material (annual, 16 participants per training) |
|  | Teacher salary | 10 hours | Hours to deliver 10, 1-hour sessions of group-based intervention per class (22 students) |
|  | Teacher salary | 2  hours | 2 hours per month for supervision of peer mentors (group of 20) |
|  | Peer mentor | 0 hours | Peer mentors are not compensated. Assume 50 peer mentors per student (20 per school) |
|  | Psychologist salary | 8 hours | Hours to conduct teacher training per school (46 teachers per school, each teacher covers 1 class of 22 students) |
|  | Psychiatrist salary | 8 hours | Training of trainers |
|  | Training materials printing | 1 | 100-page workbook printing |
|  | Mobile application* | 1 | *Mobile application free to all participants. Inclusion criteria for participating in this intervention is access to a smartphone device. |
| **Intervention** | **Ingredient** | **Units** | **Description** |
| Treatment of mild anxiety^8^ | IT infrastructure, per user | 1 | Cost of maintaining IT infrastructure^25^ |
|  | Computer | 1 | Assume useful life of 5.4 years, 1 per medical officer |
|  | Telephone call | 3 | Cost of 5-min phone call for medical officer to provide asynchronous support |
|  | Medical officer salary | .25 hours | Hours for limited support calls |
|  | Medical officer salary | 16.1 hours | Initial training to conduct intervention |
|  | Medical officer salary | 8 hours | Annual refresher training |
|  | Medical officer salary | 24 hours | 2 hours per month for supervision (annual) |
|  | Psychologist salary | 16.1 hours | Initial training of medical officers, 24 medical officers per training |
|  | Psychologist salary | 8 hours | Annual refresher training of medical officers, 24 medical officers per training |
|  | Psychologist salary | 24 hours | Supervision – 2 hours per month, group of 10 medical officers |
|  | Psychologist salary | 40 hours | Training for trainers/supervisors (initial). Includes 16 trainees. |
|  | Psychologist salary | 8 hours | Training for trainers/supervisors (annual refresher training). Includes 16 trainees. |
|  | Psychiatrist salary | 40 hours | Master trainer – training supervisors (initial) |
|  | Psychiatrist salary | 8 hours | Master trainer – training supervisors (annual) |
| **Intervention** | **Ingredient** | **Units** | **Description** |
| Treatment of moderate-severe anxiety^8^ | Medical officer salary | 12 hours | 12 1-hour CBT sessions |
|  | Medical officer salary | 16.1 hours | Initial training to conduct intervention |
|  | Medical officer salary | 8 hours | Annual refresher training |
|  | Medical officer salary | 24 hours | 2 hours per month for supervision |
|  | Psychologist salary | 16.1 hours | Delivering initial training of medical officers, 24 medical officers per training |
|  | Psychologist salary | 8 hours | Delivering annual refresher training of medical officers, 24 medical officers per training |
|  | Psychologist salary | 24 hours | Supervision – 2 hours per month, group of 10 medical officers |
|  | Psychologist salary | 40 hours | Participating in training for trainers/supervisors (initial). Includes 16 trainees. |
|  | Psychologist salary | 8 hours | Participating in training for trainers/supervisors (annual refresher training). Includes 16 trainees. |
|  | Psychiatrist salary | 40 hours | Master trainer – delivering training of supervisors (initial) |
|  | Psychiatrist salary | 8 hours | Master trainer – delivering training of supervisors (annual) |
|  | Medication (Fluoxetine) | 1 | Dosage: initially 50mg daily, then increased in steps of 5mg at intervals of at least 1 week if required; maximum 200mg per day |
| **Intervention** | **Ingredient** | **Units** | **Description** |
| Treatment of mild depression^10^ | IT infrastructure, per user | 1 | Cost of maintaining IT infrastructure^25^ |
|  | Computer | 1 | Assume useful life of 5.4 years, 1 per medical officer |
|  | Telephone call | 3 | Cost of 5-min phone call for medical officer to provide asynchronous support |
|  | Medical officer salary | .25 hours | Hours for limited support calls |
|  | Medical officer salary | 16.1 hours | Initial training to conduct intervention |
|  | Medical officer salary | 8 hours | Annual refresher training |
|  | Medical officer salary | 24 hours | 2 hours per month for supervision (annual) |
|  | Psychologist salary | 16.1 hours | Initial training of medical officers, 24 medical officers per training |
|  | Psychologist salary | 8 hours | Annual refresher training of medical officers, 24 medical officers per training |
|  | Psychologist salary | 24 hours | Supervision – 2 hours per month, group of 10 medical officers |
|  | Psychologist salary | 40 hours | Training for trainers/supervisors (initial). Includes 16 trainees. |
|  | Psychologist salary | 8 hours | Training for trainers/supervisors (annual refresher training). Includes 16 trainees. |
|  | Psychiatrist salary | 40 hours | Master trainer – training supervisors (initial) |
|  | Psychiatrist salary | 8 hours | Master trainer – training supervisors (annual) |
| **Intervention** | **Ingredient** | **Units** | **Description** |
| Treatment of moderate-severe depression^12^ | Medical officer salary | 15 hours | 12 1-hour CBT sessions, 6 30-minute medication management sessions |
|  | Medical officer salary | 24 hours | 2 hours per month for supervision |
|  | Psychiatrist salary | 24 hours | Supervision – 2 hours per month, group of 10 medical officers |
|  | Medical officer salary | 16.1 hours | Initial training to conduct intervention |
|  | Medical officer salary | 8 hours | Annual refresher training |
|  | Psychiatrist salary | 16.1 hours | Initial training of medical officers, 24 medical officers per training |
|  | Psychiatrist salary | 8 hours | Annual refresher training of medical officers, 24 medical officers per training |
|  | Fluoxetine | 84 | Daily for duration of 12-week treatment |
|  | Psychologist salary | 40 hours | Training for trainers/supervisors (initial). Includes 16 trainees. |
|  | Psychologist salary | 8 hours | Training for trainers/supervisors (annual refresher training). Includes 16 trainees. |
|  | Psychiatrist salary | 40 hours | Master trainer – training supervisors (initial) |
|  | Psychiatrist salary | 8 hours | Master trainer – training supervisors (annual) |
| **Intervention** | **Ingredient** | **Units** | **Description** |
| Treatment of bipolar disorder^15^ | Therapist salary | 16 hours | 16 60-minute sessions over 6 months |
|  | Therapist salary | 24 hours | 2 hours per month for supervision |
|  | Psychiatrist salary | 24 hours | Supervision – 2 hours per month, group of 10 medical officers. |
|  | Medical officer salary | 8 hours | Annual training to conduct intervention |
|  | Psychiatrist salary | 8 hours | Annual training of therapists, 24 therapists per training |
|  | Haloperidol | Varies | Use daily with lithium if manic episode develops. Do not use with anti-depressant. |
|  | Lithium carbonate | 365 | Daily mood stabilizer treatment for all with bipolar disorder |
|  | Fluoxetine | Varies | Use daily with lithium if depressive episode develops |
|  | Serum lithium test | First year: 4  Subsequent years: 2 | Needed when taking lithium carbonate.  Baseline, 1 week, every 3 months for 1 year, then every 6 months |
|  | Serum prolactin test | 3 | Needed when taking haloperidol.  Monthly tests for duration of manic episode (12 weeks) |
|  | Serum electrolytes test | 2 | Needed when taking haloperidol.  Every 6 months. |
|  | Serum creatinine test | 2 per year | Needed when taking lithium carbonate.  Baseline, then every 6 months |
|  | Thyroid function test | 2 per year | Needed when taking lithium carbonate.  Baseline, then every 6 months |
| **Intervention** | **Ingredient** | **Units** | **Description** |
| Universal school-based prevention of suicide^17^ | Medical officer salary | 3.25 hours | 1-hour information session and 9, 15-minute follow up contacts |
|  | Computer | 1 | Assume useful life of 5.4 years, 1 per medical officer |
|  | Phone call (per minute) | 60 | 1-hour phone call between medical officer and adolescent |
|  | Internet access (per hour) | 2.25 hours | Internet access for medical officer to conduct video calls |
|  | Medical officer salary | 15.4 hours | Initial training to conduct intervention |
|  | Medical officer salary | 8 hours | Annual refresher training |
|  | Psychologist salary | 15.4 hours | Initial training of medical officers, 24 medical officers per training |
|  | Psychologist salary | 8 hours | Annual refresher training of medical officers, 24 medical officers per training |
|  | Medical officer salary | 24 hours | Supervision – 2 hours per month |
|  | Psychologist salary | 24 hours | Supervision – 2 hours per month, group of 10 medical officers |
|  | Psychologist salary | 40 hours | Adaptation of educational materials to country, per country. Include initial adaptation then repeat every 5 years. |
|  | Psychologist salary | 40 hours | Training for trainers/supervisors (initial). Includes 16 trainees. |
|  | Psychologist salary | 8 hours | Training for trainers/supervisors (annual refresher training). Includes 16 trainees. |
|  | Psychiatrist salary | 40 hours | Master trainer – training supervisors (initial) |
|  | Psychiatrist salary | 8 hours | Master trainer – training supervisors (annual) |
| **Intervention** | **Ingredient** | **Units** | **Description** |
| Indicated prevention of suicide^18^ | Medical officer salary | 3.25 hours | 1-hour information session and 9, 15-minute follow up contacts |
|  | Computer | 1 | Assume useful life of 5.4 years, 1 per medical officer |
|  | Phone call (per minute) | 60 | 1-hour phone call between medical officer and adolescent |
|  | Internet access (per hour) | 2.25 hours | Internet access for medical officer to conduct video calls |
|  | Medical officer salary | 15.4 hours | Initial training to conduct intervention |
|  | Medical officer salary | 8 hours | Annual refresher training |
|  | Psychologist salary | 15.4 hours | Initial training of medical officers, 24 medical officers per training |
|  | Psychologist salary | 8 hours | Annual refresher training of medical officers, 24 medical officers per training |
|  | Medical officer salary | 24 hours | Supervision – 2 hours per month |
|  | Psychologist salary | 24 hours | Supervision – 2 hours per month, group of 10 medical officers |
|  | Psychologist salary | 40 hours | Adaptation of educational materials to country, per country. Include initial adaptation then repeat every 5 years. |
|  | Psychologist salary | 40 hours | Training for trainers/supervisors (initial). Includes 16 trainees. |
|  | Psychologist salary | 8 hours | Training for trainers/supervisors (annual refresher training). Includes 16 trainees. |
|  | Psychiatrist salary | 40 hours | Master trainer – training supervisors (initial) |
|  | Psychiatrist salary | 8 hours | Master trainer – training supervisors (annual) |

**Selected Consolidated Framework Implementation Research Constructs and Questions**

The ranked readiness scores used a Likert scale from 1 to 5 (lowest to highest to obtain total score per intervention).

**Table 65.** **Selected Consolidated Framework Implementation Research Constructs and Questions**

|  | **Question** | **Likert Scale = 1** | **Likert Scale =5** |
| --- | --- | --- | --- |
| 1 | How would you assess the need for this intervention? | No to minimal need | Very strong need |
| 2 | To what extent do current programs meet existing needs? | Current program fails to meet need | Current program meets all existing needs |
| 3 | How complicated is the intervention [the intervention itself – not the process of implementing it within the target setting]? Please consider the following aspects of the intervention: duration, scope, intricacy, and whether the intervention reflects a clear departure from previous practices. | Very complicated | Very simple |
| 4 | To what extent are changes or alterations needed to make to the intervention work effectively in the target setting? | Many changes needed | No changes needed |
| 5 | How well does the intervention fit with existing work processes and practices in the target setting? | Intervention does not fit into existing process and practices | Intervention fits very well into existing processes and practices |
| 6 | How will the infrastructure of the target setting (social architecture, age, maturity, size, or physical layout) affect the implementation of the intervention? | Infrastructure will significantly hinder implementation; | Infrastructure will significantly facilitate implementation |
| 7 | How do you think the target setting’ culture (general beliefs, values, assumptions that people embrace) will affect the implementation of the intervention? | Very adversely influence implementation | Very positively influence implementation |
| 8 | What may be the general level of receptivity in the target setting to implementing the intervention? | Very low | Very high |
| 9 | What may be the priority of getting the intervention implemented relative to other initiatives that are happening now? | Very low priority | Very high priority |
| 10 | What level of endorsement or support will there be from leaders within the setting? | No endorsement or support | Strong and wide-spread endorsement or support |

**Model Parameter Values**

The model parameter values and data sources can be found in the supplementary materials Excel file, “Supplemental file parameter values.”

- Parameter and ranges
- Starting population
- Monthly incidence of conditions/events of interest and mortality
- Education and employment statistics by age and sex
- Unit costs
- Total monthly intervention costs per person treated per month

**Sensitivity Analysis**

To account for uncertainty, a probabilistic sensitivity analysis (PSA) was conducted to generate a 90 percent uncertainty interval (90% UI) for our outcomes of interest.^40^ For each intervention or set of interventions, it conducted 1,000 runs of the model using a Latin hypercube sampling approach to generate independently randomly selected values for each of our parameters along its distribution. From the results of those 1,000 runs, it generated the 90% UI via the percentile method.

To compare the difference in outcomes by sex, the study conducted a linear mixed effects analysis of the effect of sex on the outcome of interest that included sex as a fixed effect plus a random effect for hypercube sample row to control for repeated measures of the same PSA sample. The study then conducted a likelihood ratio test comparing this model with a model of only the random effects by hypercube sample row; the results were considered significantly different by sex at a level α = 0.05.

**Supplementary Results**

**Table S7. Annual Cost per Adolescent who Received an Intervention and Share of Annual NHS England Spending Per Capita (2021-2022).**

| **Intervention** | **Annual cost per adolescent who received the intervention** (2021 GBP) | | **Share of annual public health funding per capita**  (2021 GBP 2,409 per capita)^41^ | |
| --- | --- | --- | --- | --- |
|  |  |  |  |  |
|  | First Year | Subsequent Years | First Year | Subsequent Years |
| Universal Prevention of Anxiety and Depression | 65 | 64 | 2.7% | 2.7% |
| School-based Suicide Prevention | 16 | 1 | 0.7% | 0.1% |
| Hospital-based Indicated Suicide Prevention | 118 | 114 | 4.9% | 4.7% |
| Treatment of Mild Anxiety | 142 | 9 | 5.9% | 0.4% |
| Treatment of Moderate and Severe Anxiety | 3,223 | 3,057 | 133.8% | 126.9% |
| Treatment of Mild Depression | 121 | 121 | 5.0% | 5.0% |
| Treatment of Moderate and Severe Depression | 1,640 | 1,642 | 68.1% | 68.2% |
| Treatment of Bipolar Disorder | 176 | 171 | 7.3% | 7.1% |

**Table S8. Readiness Scores and Cumulative Ranking by Intervention**

|  | **Q1** | **Q2** | **Q3** | **Q4** | **Q5** | **Q6** | **Q7** | **Q8** | **Q9** | **Q10** | **Cumulative Score**  (Highest score denotes intervention is easier to implement) | **Ranking** |
| --- | --- | --- | --- | --- | --- | --- | --- | --- | --- | --- | --- | --- |
| **Intervention** | How would you assess the need for this intervention? | To what extent do current programs meet existing needs? | How complicated is the intervention [the intervention itself – not the process of implementing it within the target setting]? | To what extent are changes or alterations needed to make to the intervention work effectively in the target setting? | How well does the intervention fit with existing work processes and practices in the target setting? | How will the infrastructure of the target setting (social architecture, age, maturity, size, or physical layout) affect the implementation of the intervention? | How do you think the target setting’ culture (general beliefs, values, assumptions that people embrace) will affect the implementation of the intervention? | What may be the general level of receptivity in the target setting to implementing the intervention? | What may be the priority of getting the intervention implemented relative to other initiatives that are happening now? | What level of endorsement or support will there be from leaders within the setting? |  |  |
| Hospital-based indicated prevention of suicide | 46 | 24 | 37 | 33 | 35 | 33 | 30 | 31 | 36 | 37 | **342** | **1** |
| Universal school-based prevention of suicide | 40 | 24 | 37 | 36 | 33 | 33 | 30 | 33 | 32 | 35 | **333** | **2** |
| Universal prevention of anxiety and depression | 42 | 24 | 36 | 34 | 37 | 37 | 30 | 30 | 26 | 36 | **332** | **3** |
| Treatment of moderate and severe anxiety | 41 | 21 | 32 | 23 | 38 | 35 | 30 | 37 | 31 | 42 | **330** | **4** |
| Treatment of mild anxiety | 40 | 23 | 37 | 34 | 26 | 34 | 31 | 31 | 30 | 34 | **320** | **5** |
| Treatment of mild depression | 36 | 24 | 39 | 35 | 32 | 31 | 28 | 32 | 27 | 32 | **316** | **6** |
| Treatment of moderate and severe depression | 40 | 21 | 24 | 27 | 32 | 35 | 31 | 31 | 29 | 36 | **306** | **7** |
| Treatment of bipolar disorder | 40 | 22 | 23 | 27 | 31 | 29 | 32 | 33 | 30 | 35 | **302** | **8** |

**Table S9** summarises the results from the investment case and implementability analyses by ranking each intervention based on health, economic, implementation readiness, and affordability. The school-based anxiety and depression prevention intervention not only offered the highest number of health and net economic benefits, but was among the more affordable, implementation-ready interventions. In contrast, whereas suicide prevention interventions were considered the most implementation-ready and more affordable, their relative health and economic effects were the lowest among the interventions.

**Table S9. Intervention Ranking by Health, Economic, and Implementability Indicators Over a Lifetime Time-Horizon**

|  | **Health Indicators** | **Economic Indicators**  **(80-year Time Horizon)** | | | **Implementability Indicators** | |
| --- | --- | --- | --- | --- | --- | --- |
|  | DALYs Averted | ROI | Cost per DALY averted | Net Benefits | Affordability | Readiness |
| Mild Anxiety Treatment | 2 | 1 | 1 | 2 | 2 | 5 |
| School-based Anxiety and Depression Prevention | 1 | 3 | 3 | 1 | 3 | 3 |
| Mild Depression Treatment | 3 | 2 | 2 | 3 | 5 | 6 |
| Moderate-Severe Depression Treatment | 4 | 4 | 4 | 4 | 7 | 7 |
| Bipolar Disorder Treatment | 5 | 5 | 5 | 5 | 6 | 8 |
| Moderate-Severe Anxiety Treatment | 6 | 8 | 8 | 6 | 8 | 4 |
| School-based Suicide Prevention | 7 | 7 | 7 | 7 | 1 | 2 |
| Hospital-based Suicide Prevention | 8 | 6 | 6 | 8 | 4 | 1 |

*Ranking by color: green (ranked 1^st^ or 2^nd^); blue (ranked 3^rd^ or 4^th^); yellow (ranked 5^th^ or 6^th^); or red (ranked 7^th^ or 8^th^).*

**References**

1. Crenna-Jennings W, Hutchinson J. *Access to Child and Adolescent Mental Health Services in 2019*.; 2020. https://epi.org.uk/publications-and-research/access-to-child-and-adolescent-mental-health-services-in-2019/. Accessed September 19, 2022.

2. England E, Mughal F. Underprovision of mental health services for children and young people. *Br J Gen Pract*. 2019;69(680):112-113. doi:10.3399/bjgp19X701381

3. Frith E. Children and Young People’s Mental Health: State of the Nation: April 2016. http://dera.ioe.ac.uk/27926/1/State-of-the-Nation-report-web.pdf. Published April 2016. Accessed November 30, 2022.

4. NHS England. *The NHS Long Term Plan*.; 2019. https://www.longtermplan.nhs.uk/publication/nhs-long-term-plan/. Accessed September 19, 2022.

5. Punukollu M, Burns C, Marques M. Effectiveness of a pilot school-based intervention on improving scottish students’ mental health: a mixed methods evaluation. *International Journal of Adolescence and Youth*. 2020;25(1):505-518. doi:10.1080/02673843.2019.1674167

6. Punukollu M, Leighton EL, Brooks AF, et al. SafeSpot: an innovative app and mental health support package for Scottish schools – a qualitative analysis as part of a mixed methods study. *Child Adolesc Ment Health*. 2020;25(2):110-116. doi:10.1111/camh.12375

7. Stockings EA, Degenhardt L, Dobbins T, et al. Preventing depression and anxiety in young people: a review of the joint efficacy of universal, selective and indicated prevention. *Psychological Medicine*. 2016;46(1):11-26. doi:10.1017/S0033291715001725

8. National Institute for Health and Care Excellence. Anxiety disorders: Quality Standards [QS53]. https://www.nice.org.uk/guidance/qs53. Published February 6, 2014. Accessed August 8, 2023.

9. James AC, James G, Cowdrey FA, Soler A, Choke A. Cognitive behavioural therapy for anxiety disorders in children and adolescents. *Cochrane Database of Systematic Reviews*. 2015;(2). doi:10.1002/14651858.CD004690.pub4

10. Abeles P, Verduyn C, Robinson A, Smith P, Yule W, Proudfoot J. Computerized CBT for adolescent depression (“Stressbusters”) and its initial evaluation through an extended case series. *Behav Cogn Psychother*. 2009;37(2):151-165. doi:10.1017/S1352465808005067

11. Oud M, de Winter L, Vermeulen-Smit E, et al. Effectiveness of CBT for children and adolescents with depression: A systematic review and meta-regression analysis. *Eur Psychiatry*. 2019;57:33-45. doi:10.1016/j.eurpsy.2018.12.008

12. National Institute for Health and Care Excellence. Depression in children and young people: identification and management NICE guideline [NG134]. https://www.nice.org.uk/guidance/ng134/chapter/Recommendations#steps-4-and-5-managing-moderate-to-severe-depression. Published June 25, 2019. Accessed August 8, 2023.

13. Domino ME, Burns BJ, Silva SG, et al. Cost-Effectiveness of Treatments for Adolescent Depression: Results From TADS. *AJP*. 2008;165(5):588-596. doi:10.1176/appi.ajp.2008.07101610

14. Kennard B, Silva S, Vitiello B, et al. Remission and residual symptoms after short-term treatment in the Treatment of Adolescents with Depression Study (TADS). *J Am Acad Child Adolesc Psychiatry*. 2006;45(12):1404-1411. doi:10.1097/01.chi.0000242228.75516.21

15. Sharma A, Glod M, Forster T, et al. FAB: First UK feasibility trial of a future randomised controlled trial of Family focused treatment for Adolescents with Bipolar disorder. *International Journal of Bipolar Disorders*. 2020;8(1):24. doi:10.1186/s40345-020-00189-y

16. Miklowitz DJ, Axelson DA, Birmaher B, et al. Family-Focused Treatment for Adolescents With Bipolar Disorder: Results of a 2-Year Randomized Trial. *Arch Gen Psychiatry*. 2008;65(9):1053-1061. doi:10.1001/archpsyc.65.9.1053

17. Wasserman D, Hoven CW, Wasserman C, et al. School-based suicide prevention programmes: the SEYLE cluster-randomised, controlled trial. *Lancet*. 2015;385(9977):1536-1544. doi:10.1016/S0140-6736(14)61213-7

18. Fleischmann A, Bertolote JM, Wasserman D, et al. Effectiveness of brief intervention and contact for suicide attempters: a randomized controlled trial in five countries. *Bull World Health Organ*. 2008;86(9):703-709. doi:10.2471/BLT.07.046995

19. NHS. Coventry and Warwickshire Area Prescribing Committee Formulary - Formulary Chapter 4: Nervous system 04.03 (Oct 2018 version 7.1). https://www.covwarkformulary.nhs.uk/chaptersSub.asp?FormularySectionID=4. Published May 2019. Accessed August 8, 2023.

20. Management Sciences for Health. International Medical Products Price Guide. 2016. https://www.msh.org/resources/international-medical-products-price-guide.

21. *MhGAP Intervention Guide for Mental, Neurological and Substance Use Disorders in Non-Specialized Health Settings*. World Health Organization; Mental Health Gap Action Programme; 2016. http://apps.who.int/iris/bitstream/10665/250239/1/9789241549790-eng.pdf?ua=1. Accessed June 2, 2017.

22. Masila C. Query about lab costs for NCDs. 2020.

23. International Labor Organization. Statistics on labour costs - ILOSTAT. https://ilostat.ilo.org/topics/labour-costs/. Accessed August 8, 2023.

24. Ahern S, Apter A, Balazs J, et al. A cost-effectiveness analysis of school-based suicide prevention programmes. *European Child & Adolescent Psychiatry*. 2018;27(10):1295-1304. doi:10.1007/s00787-018-1120-5

25. Jolstedt M, Ljótsson B, Fredlander S, et al. Implementation of internet-delivered CBT for children with anxiety disorders in a rural area: A feasibility trial. *Internet Interv*. 2017;12:121-129. doi:10.1016/j.invent.2017.11.003

26. Bureau of Transportation Statistics. *Transportation and Economic Trends 2018*. Washington D.C.: United States Department of Transportation; 2018. https://doi.org/10.21949/1502599.

27. National Sample Survey Office. *Key Indicators of Household Expenditure on Services and Durable Goods Survey*. New Delhi: Ministry of Statistics and Programme Implementation; 2016. https://www.thehinducentre.com/multimedia/archive/02914/Key_Indicators_of__2914425a.pdf.

28. Paiva G, Silva D, Feijo C. *Consumption and Socioeconomic Classification in Brazil: A Study Based on the Brazilian Family Expenditure Survey*.; 2013.

29. Household Expenditure Survey, Australia: Summary of Results, 2015-16 financial year | Australian Bureau of Statistics. https://www.abs.gov.au/statistics/economy/finance/household-expenditure-survey-australia-summary-results/latest-release. Published September 13, 2017. Accessed January 26, 2021.

30. Statistics South Africa. *Measuring Household Expenditure on Public Transport: In-Depth Analysis of the National Household Travel Survey 2013 Data*. Pretoria, South Africa: Statistics South Africa; 2015. http://www.statssa.gov.za/publications/Report-03-20-11/Report-03-20-112013.pdf.

31. Ethiopia Central Statistical Agency. *The 2015/16 Ethiopian Household Consumption - Expenditure (HCE) Survey*. Addis Ababa: Ethiopia Central Statistical Agency; 2018.

32. Alliance for Affordable Internet. 2022 prices and affordability of smartphones and feature phones by country [database]. https://a4ai.org/research/device-pricing-2022/. Published August 31, 2022. Accessed August 8, 2023.

33. International Telecommunications Union. *Measuring Digital Development: ICT Price Trends 2019*. ITU; 2020. https://www.itu.int/en/mediacentre/Documents/Documents/ITU-Measuring_Digital_Development_ICT_Price_Trends_2019.pdf.

34. Lee YY, Barendregt JJ, Stockings EA, et al. The population cost-effectiveness of delivering universal and indicated school-based interventions to prevent the onset of major depression among youth in Australia. *Epidemiology and Psychiatric Sciences*. 2017;26(5):545-564. doi:10.1017/S2045796016000469

35. World Bank. World Development Indicators. DataBank. http://databank.worldbank.org/data/reports.aspx?source=world-development-indicators. Published June 1, 2017. Accessed June 22, 2017.

36. Chisholm D, Sweeny K, Sheehan P, et al. Scaling-up treatment of depression and anxiety: a global return on investment analysis. *The Lancet Psychiatry*. 2016;3(5):415-424. doi:10.1016/S2215-0366(16)30024-4

37. Chang AY, Robinson LA, Hammitt JK, Resch SC. Economics in “Global Health 2035”: a sensitivity analysis of the value of a life year estimates. *J Glob Health*. 2017;7(1):010401. doi:10.7189/jogh.07.010401

38. Stenberg K, Axelson H, Sheehan P, et al. Advancing social and economic development by investing in women’s and children’s health: a new Global Investment Framework. *Lancet*. 2014;383(9925):1333-1354. doi:10.1016/S0140-6736(13)62231-X

39. Jamison DT, Summers LH, Alleyne G, et al. Global health 2035: a world converging within a generation, Supplementary appendix 3. *The Lancet*. 2013;382(9908):1898-1955. doi:10.1016/S0140-6736(13)62105-4

40. Stelmach R, Kocher EL, Kataria I, Jackson-Morris AM, Saxena S, Nugent R. The global return on investment from preventing and treating adolescent mental disorders and suicide: a modelling study. *BMJ Global Health*. 2022;7(6):e007759. doi:10.1136/bmjgh-2021-007759

41. World Bank. World Development Indicators. https://databank.worldbank.org/source/world-development-indicators. Published 2022.
